# A Bridge Between Cultures for Better Patient Care: A Qualitative Study of the Nurse‐Led Rapid Response Team

**DOI:** 10.1111/scs.70064

**Published:** 2025-07-02

**Authors:** Tóra Róin, Matilde S. Axelsen, Gitte Bunkenborg

**Affiliations:** ^1^ Department of Cardiology Zealand University Hospital Roskilde Denmark; ^2^ Research Support Unit Zealand University Hospital Roskilde Denmark; ^3^ Department of Clinical Research University of Southern Denmark Odense Denmark; ^4^ Department of Medicine 2 Holbaek Hospital Holbaek Denmark; ^5^ Department of Anesthesiology Holbaek Hospital Holbaek Denmark; ^6^ Department of Regional Health Research University of Southern Denmark Odense Denmark

**Keywords:** communication, end‐of‐life care decision‐making, hospital rapid response team, nursing, patient care, teaching

## Abstract

**Introduction:**

Rapid Response Teams (RRTs) are crucial for patient safety, yet their effectiveness hinges on organisational factors, clinical culture, inter‐professional collaboration and ward nurse attitudes. Understanding ward nurses' perceptions is vital for optimising RRT impact on patient care and safety culture.

**Aims:**

This study aimed to explore general ward registered nurses' perceptions of nurse‐led RRTs, focusing on their collaborative experiences and reasons for team activation.

**Methods:**

A qualitative exploratory design was employed. Focus group interviews were conducted with 22 general ward nurses across three Danish hospitals, recruited via purposive sampling. A semi‐structured interview guide facilitated discussions. Interpretive content analysis with a hermeneutical approach was used to identify key themes.

**Results:**

Participants expressed a predominantly positive perception of RRTs, encapsulated in the core theme: ‘A vital and dynamic resource, benefitting patient care and nursing practice’. Key categories included: ‘Perceiving patient safety as the main purpose of RRT’, ‘Supporting nurses' sense of security’, ‘Delivering practical assistance and teaching’, ‘Providing patient‐centred care’ and ‘Fostering constructive communication and collaboration’. Nurses found experienced physicians supportive but expressed greater confidence in RRT‐nurses compared to junior physicians. Frustration with a lack of physician involvement in treatment limitation and end‐of‐life care discussions was also noted.

**Conclusions:**

Nurse‐led RRTs are valued by ward nurses, providing essential support and bridging multidisciplinary collaboration and communication. However, limitations in the RRT's teaching role suggest a need for supplementary educational strategies. Shared decision‐making regarding treatment limitation and end‐of‐life care is crucial for maximising RRT benefits.

**Limitations:**

Findings may have limited transferability due to the study's specific healthcare context.

## 1 Introduction

Despite ongoing efforts to improve patient safety culture, hospitals face persistent challenges in the implementation and long‐term sustainability of structured patient safety interventions [1, 2]. Effective management of deteriorating patients is a patient safety concern and relies heavily on ward nurses' clinical observation, judgment and timely intervention [3]. Nurses are pivotal in initiating care escalation, including Rapid Response Team (RRT) activation, when patients exhibit signs of acute deterioration, such as an elevated Early Warning Score (EWS) [3, 4, 5]. While standard procedures often mandate RRT activation based on algorithm‐based EWS systems [6, 7], nurses' concerns also trigger these responses. However, inconsistent adherence to EWS protocols among nursing staff remains a significant barrier to timely RRT activation [8, 9]. To optimise patient care, safety culture and nursing resource utilisation, RRT development must prioritise patient‐centred care while addressing nurses' needs and overcome contextual barriers [10]. Notably, geographical and cultural factors influence healthcare professional collaboration concerning RRT activation [11]. Recognising the significance of context is therefore essential to maintain effective RRTs across a variety of hospital settings.

## 2 Background

RRTs play a vital role in enhancing patient safety through rapid assessment and stabilisation of deteriorating patients [12, 13, 14].

While RRTs generally include critical care professionals, their structural composition differs among hospital facilities. To maximise the benefits of RRTs in improving patient care and optimising resource utilisation, effective implementation, informed by contextual factors, is essential [15, 16, 17, 18].

Numerous barriers and facilitators influence RRT activation. These include organisational factors, hospital infrastructure, clinical culture and nurses' individual attributes, such as knowledge and experience [14, 15, 16, 17, 19]. Specifically, nurses may experience uncertainty regarding activation criteria, concerns about increased workload, negative prior experiences, knowledge deficits and the perceived need to justify RRT calls [20, 21, 22]. The dynamics of interactions between nurses and RRT members can be complex, presenting both challenges and opportunities. While communication barriers may impede timely activation, collaborative interactions can also foster nurses' clinical skill development [23]. Workplace culture, including interprofessional collaboration and hierarchical structures, significantly influences timely RRT activation [11]. Furthermore, effective physician‐nurse collaboration within physician‐staffed RRTs/Medical Emergency Teams (METs) has been shown to positively impact patient safety culture [24].

While quantitative research on RRT effectiveness is abundant, qualitative exploration of nurses' perceptions is crucial to provide a deeper understanding and inform future RRT development. Although recent qualitative research has explored nurses' perspectives on the Critical Care Outreach Team in Ireland, a similar service, studies employing qualitative methodologies to delve into general ward nurses' perceptions of using and collaborating with nurse‐led RRTs remain scarce [25]. This gap in the literature highlights the need for further qualitative research to explore the nuanced experiences of ward nurses interacting with nurse‐led RRTs. Therefore, this study explores general ward registered nurses' perceptions of calling and collaborating with nurse‐led RRTs across three Danish hospitals.

### 2.1 Aim

This study aimed to explore general ward nurses' perceptions of calling and collaborating with the nurse‐led RRT.

## 3 Methods

### 3.1 Design

This study employed a qualitative design leaning on a hermeneutic approach in an interpretive content analysis of focus group discussions with ward nurses' perceptions of calling and collaborating with the nurse‐led RRT [26, 27]. Gadamer argues that understanding is not a purely objective process but rather a dynamic interaction between the interpreter and, in our case, the ward nurses' narratives [28]. This interaction is shaped by the interpreter's pre‐understandings, or ‘horizons of understanding’, which are influenced by their cultural, historical and personal experiences [28].

### 3.2 Reflections and Pre‐Understandings

As researchers, our pre‐understandings and interpretations are influenced by our educational, training and clinical backgrounds. The last author, an ICU‐trained female nurse with a research interest in RRTs, moderated the focus groups. Her RRT experience was limited and dated to over 15 years prior to the commencement of this research project. The other two authors, registered nurses with master's degrees, were receiving research training prior to PhD studies at the time of data collection. They both have some RRT activation experience from years back. The researchers acknowledge that their varied clinical and research backgrounds, including the first author's dated RRT experience and the other authors' research training, could have influenced their epistemological positioning and reflexivity. Throughout the research process, the authors engaged in open reflexive discussions to critically examine and address their pre‐understandings.

### 3.3 Setting

The study was conducted in three acute care hospitals situated in different regions of Denmark. One was a 750‐bed university hospital, while the other two were 330‐bed university‐affiliated hospitals. All three institutions had a nurse‐led RRT operating and providing round‐the‐clock coverage to adult patients on medical and surgical wards for over a decade.

Denmark operates a universal healthcare system primarily funded through taxation, ensuring equal access to healthcare services for all citizens. The system is characterised by publicly funded hospitals and primary care, organised regionally. A strong emphasis is placed on prevention and public health. The nurse‐led RRT was introduced nationwide approximately 10–15 years ago as a vital component of Danish healthcare and patient safety.

#### 3.3.1 The Rapid Response Team

Across the three study sites, nurse‐led RRTs operated with a consistent core structure. A critical care‐trained RRT nurse responded to activation calls from ward nurses, conducting initial patient assessments. In cases requiring medical intervention, the RRT nurse consulted with an on‐call ICU consultant prior to departing the ICU. A dedicated RRT nurse at each site oversaw team operations, including nurse training, data collection and collaboration with ward managers. However, the scope of RRT nurse responsibilities varied significantly. At one site, RRT duties were integrated with ICU patient care, without additional compensation. Conversely, at the other two sites, RRT nurses focused primarily on RRT and administrative tasks, receiving supplementary financial compensation.

### 3.4 Participants and Sampling

A purposive sampling strategy was employed to recruit participants with diverse characteristics, encompassing age, sex, years of nursing experience and ward type (medical or surgical). Eligible participants were required to be registered nurses with prior experience in both activating RRTs and participating in RRT reviews. General ward nurses were recruited through open invitations distributed in their lunchrooms, encouraging participation from nurses of all ages, genders, experience levels and from both surgical and medical departments. Managers facilitated participation by arranging coverage for patient responsibilities during interview sessions but had no influence on participant selection. To encourage open dialogue and minimise potential influence from existing ward relationships, participants from the same ward were not included in the same focus group, and efforts were made to include participants who were not previously well acquainted.

### 3.5 Data Collection

Focus group interviews were conducted in hospital offices between September 2018 and March 2019, following ward nurses' day shifts.

The discussions were moderated by an experienced qualitative postdoctoral researcher (last author) using a semi‐structured interview guide. The guide was developed based on existing literature and collaborative discussions within the research team (Table 1). Each session commenced with open‐ended questions: ‘Why do you think this hospital has an RRT?’ and ‘Can you recall your first experience of calling the RRT?’ Subsequent discussions were guided by prompts outlined in Table 1, aiming to address the research question by exploring four specific areas: participants' experiences and perceptions of their roles and the RRT‐nurse's role during RRT reviews, collaborative dynamics between healthcare professionals, the perceived patient perspective, and organisational factors influencing the delivery of RRT services. An observer was present ensuring equal participation and taking field notes. All focus groups were digitally recorded.

**TABLE 1:** Interview guide on general ward nurses' perceptions of a nurse‐led RRT.

Two opening questions Why, in your opinion, is there a nurse‐led RRT in this hospital? Do you remember the first time you called the team?			
**Four areas of interest to be discussed during the focus group sessions**			
The role of the general ward nurse in RRT‐calls	The role of the RRT‐ nurse	The patient perspective	Organisational issues
**Examples of questions and probes to guide the discussions**			
How do you experience RRT‐calls? What considerations do you make before calling the RRT? Are there situations where you have chosen not to call? Why was that? What expectations do you have of the RRT and the service they provide? Do you feel a need to align expectations with the RRT‐nurse? Does your experience as a nurse influence your engagement with the RRT? What does it mean to nurses in general wards that a RRT is at hand? How do you see that? Do nurses share a mutual understanding of the RRT? Can you describe how? How do you experience the teamwork around a call?	What was originally the main role and task of RRT? Today, in your understanding, what is the most important task/role of the team? Does the RRT deliver an educational task? How does the RRT‐nurse interact with you? How do you think about the RRT‐nurse asking questions about Limitations‐of‐Medical‐Treatment?	How are the patient's needs and interests taken care of during the RRT‐ call? Does the RRT care for patients' ethical and communicative needs? What significance does RRT have to the patient?	What are your reflections about RRT and interdisciplinary collaboration? What, in your opinion, does the hospital gain from having a RRT? What is currently the biggest obstacle to a well‐functioning RRT in your hospital? Could something be handled differently?

Participants had no working relation to the researchers and were unaware of the female moderator's research interests prior to study commencement. Participants were informed about her background as a researcher in nursing. To determine data saturation, the last two interviews were carefully analysed for emergent themes. Data collection ceased when no new information was identified.

### 3.6 Data Analysis

Interview transcripts were analysed using an inductive, interpretive content analysis approach inspired by Graneheim and Lundman. This approach includes phases of de‐contextualization and re‐contextualization and processes of abstraction and interpretation, reflecting the hermeneutical approach in the analysis [26, 29, 30]. Recordings were transcribed verbatim by the first author who also consulted and incorporated the field notes taken during interviews. The first and second authors independently coded the entire dataset to enhance trustworthiness, identifying and condensing meaning units and assigning codes closely aligned with the text. They subsequently met to discuss and resolve any discrepancies in their coding, achieving complete agreement. The last author was available for consultation in the event of unresolved differences, but this proved unnecessary. In an initial interpretive process, the first author sorted codes into subcategories and categories, representing the manifest and primarily descriptive content. These were then discussed and refined collaboratively with the second and last author, and in an interpretive process of moving back and forth, looking at the text material as a whole and at the individual focus group discussions, in a hermeneutical circular process, a higher level of abstraction was applied to deepen the analysis. This process led to the formulation of an overarching theme, the underlying red thread running through the categories, representing the interpretive latent content [26, 30]. Table 2 presents an example of the coding tree. Reflexivity was maintained throughout the analysis process through open discussions about data interpretation, preventing superficial descriptions and fostering a dialogical engagement between researchers' pre‐understandings and the study's focus.

**TABLE 2:** An example of the coding tree in a qualitative content analysis of general ward nurses' perceptions of the nurse‐led RRT.

	Condensed meaning unit/codes	Sub‐category	Category
**Meaning unit focus group interview no. 7**			
S2: I think there is some learning in being with a RRT, or with a RRT‐nurse who comes up. You yourself were the first person to see the patient and you came up with an assessment and reflections regarding what could this be about, and then what you had been thinking was actually right. That is an important part. It [The RRT‐ call] supports you and prepares you for when you are in a new situation. Then you feel more confident and that helps developing your skills and competences I: *So you learn things that you can use in a new situation that might resemble the one you just managed with the RRT‐nurse?* S2: Yes, and maybe it also strengthens the self‐confidence of young nurses (If the nurse calling the RRT tell the RRT‐nurse) ‘I've seen this and that’ and the RRT‐nurse says it's correct… then it must help strengthening the self‐confidence in a similar situation… and I think that's needed … self‐confidence	One learns something being in a RRT‐call RRT‐nurses acknowledge ward nurses assessments and reflections It provides support for handling patient deteriorates It supports professional development RRT strengthens self‐confidence in young nurses Acknowledgement from RRT gives self‐confidence	Learning possibilities in RRT Enhancing competences and skills Development Provides self‐confidence	Delivering practical assistance and teaching Supporting nurses' sense of security
**Meaning unit focus group interview no. 4**			
S1: A barrier for calling lies within us. They are always positive and they would say: ‘Thank you for calling – it was the right thing to do’. Well, they don't always say it just like that, but they are accommodating and they really want to help *I: What about among you nurses? What happens when you tell your colleagues that you called the RRT the other night? What would a typical remark to that sound like?* S1: Well, it….they all think it is a god thing that we have the RRT S3: I have never heard anything negative or the like S1: No, no nothing like ‘Oh, so you could not handle it yourself!’ S3: No [on the contrary] our colleagues would say: ‘I certainly understand why you called’ Or they would express that they understood. I think the reason why we may hesitate to call and need to be reassured by a colleague is that one chooses to make the decision to call without necessarily involving the physician	RRT is always positive and accommodating RRT wants to help We all believe RRT is a good resource Never a negative remark The nursing staff appreciates RRT No indications of negative comments There is a collegial understanding for the need to call RRT Needs colleagues' ‘informal’ approval because decision to call RRT does not involve the physician	Positive Accommodating Helping The barrier is within yourself Nursing staff pro RRT No negative remarks No indications of non‐support Mutual understanding in the need for RRT Needs approval from colleagues Calls RRT without involving physician	Supporting nurses' sense of security Supporting nurses' sense of security Fostering constructive communication and collaboration Supporting nurses' sense of security

### 3.7 Rigour

To ensure research rigour, this study addressed the four trustworthiness criteria outlined by Lincoln and Guba [31]: credibility, confirmability, dependability and transferability. Researcher triangulation during data analysis enhanced credibility and confirmability. Rich data collection, including in‐depth focus group discussions and detailed field notes, was employed to ensure study dependability. By providing descriptions of the study setting and participants, and by representing diverse perspectives within the findings, transferability of results to other contexts was strengthened. Due to the lack of personal participant information and high staff turnover in hospital wards, participant feedback on study findings was not obtained.

The reporting of this study adheres to the Consolidated Criteria for Reporting Qualitative Research (COREQ) guidelines [32].

### 3.8 Ethical Considerations

The study adhered to the principles outlined in the Declaration of Helsinki [33]. According to national research regulations, ethical approval by a formal ethics committee is not required for qualitative studies.

Written informed consent was obtained from all participants prior to their involvement. Participants were assured of data confidentiality, the right to withdraw from the study at any time, and complete anonymity. The study adhered to all relevant data protection regulations, and hospital managers at the three participating hospitals provided approval.

## 4 Findings

The study involved eight focus groups with 22 female nurses from general medical and surgical wards. Due to a higher number of medical wards and staff, a greater proportion of participants came from medical wards. Reflecting the predominantly female demographic of bed ward nurses in Denmark, no male nurses responded to the invitation or were recruited for this study. The composition of the focus groups varied due to practical constraints and participant availability, resulting in small sized groups. While ideally a consistent larger group size would have been preferred, the primary objective was to gather diverse perspectives from nurses across different wards [34]. Therefore, the final number of participants in each group was determined by the number of nurses who were available and willing to participate on the scheduled dates.

Participants had a median age of 28 years and a median nursing experience of 3.75 years. Focus group sessions, detailed in Table 3, ranged in duration from 39 to 65 min (mean: 52 min). Detailed participant characteristics is presented in Table 4.

**TABLE 3:** Participation and duration of focus groups interviews.

Hospital setting	Focus group interview ID no.	Number of participants at each focus group (*n* 22)	Duration in minutes
A	1 and 2	3 and 3	59 and 65
B	3 and 4	2 and 2	39 and 40
C	5, 6, 7 and 8	4, 3, 3 and 2	56, 47, 54 and 52

**TABLE 4:** Participant characteristics.

Focus groups, *n*	8
Gender	
Male, *n*	0
Female, *n*	22
Age in years, median (range)	28 (25–57)
Years of professional experience, median (range)	3.75 (1–33)
Surgical unit, *n*	7
Medical unit, *n*	15

Analysis of focus group transcripts revealed a predominantly positive perception of calling and collaborating with the nurse‐led RRT, characterised by respect and satisfaction. A core theme was identified entitled: ‘A vital and dynamic resource, improving patient care and nursing practice’ This theme reflects profound respect for RRT nurses, who are perceived as providing essential clinical, communicative, teaching support to patients, relatives and nursing staff. Five primary categories, supported by 48 subcategories, underpinned this theme: ‘Perceiving patient safety as the main purpose of RRT’, ‘Supporting nurses' sense of security’, ‘Delivering practical assistance and teaching’, ‘Providing patient‐centred care’ and ‘Fostering constructive communication and collaboration’. An overview of the study findings is presented in Table 5.

**TABLE 5:** Findings.

Theme	A vital and dynamic resource, improving patient care and nursing practice				
Categories	Perceiving patient safety as the main purpose of RRT	Supporting nurses' sense of security	Delivering practical assistance and teaching	Providing patient‐ centred care	Fostering constructive communication and collaboration
Sub‐categories	Preventing intensive care referrals Preventing hospital cardiac arrest, and complications Delivering assistance, advice and guidance Reducing mortality Saving resources Limitless assistance and support Providing expert clinical support Experts in circulatory and respiratory problems Managing acute physiological deterioration	Imposing self‐confidence in ward nurses Create calm in chaos Friendly despite stressful situations Security when ward nurses worry Ward nurses rely heavily on the RRT RRT‐ a safety net RRT‐ a lifeline RRT‐ are indispensable RRT never leaving ward nurses behind Instant assistance Caring and professional Safe place for ward nurses to express uncertainty RRT‐nurses are positive, humble and considered	Technical expertise Basic practical help in less urgent situations Bedside teaching and learning Developing new skills via RRT‐ calls Receiving advice and support Needing structured teaching Enhancing self‐confidence through learning Contributing to developing skills New insights to less‐experienced ward nurses	Involving patient and relatives Engaging in end‐of‐life care decisions Improving patient care Always focusing on the patient perspective Facilitating timely, patient‐centred decision‐making Decision‐making regarding treatment limitation RRT facilitates timely, intensive care referrals	Constructive coaching Attentive and appreciative team‐work Addressing interprofessional communication challenges on general wards Strengthens collaboration and communication Facilitates end‐of‐life care and treatment limitation discussions with physicians Providing clinical guidance and expertise Physicians often rely on the RRTs expertise and recommendations when making critical care decisions Senior physicians are supportive of RRT Junior physicians often hesitate to call RRT Communication barriers between junior physicians and ward nurses

### 4.1 Perceiving Patient Safety as the Main Purpose of RRT

Ward nurses primarily perceived the RRT as a critical resource for stabilising acutely deteriorating patients and preventing cardiac arrest. Assistance, counselling and guidance were consistently mentioned as core functions. While nurses generally regarded RRT nurses as experts in managing critically ill patients, there was variability in how the team's purpose was defined and the circumstances prompting RRT activation, such as using the EWS. Notably, ward nurses indicated that there were no explicit limitations on when or why to call the RRT. Ward nurses described the RRT's purpose as providing help no matter where patients are situated, stabilising unstable patients and accommodating patient needs, stating that the RRT is called when patient needs exceed available ward resources. This could include circulatory or respiratory issues, staff shortages, or feelings of insecurity, particularly among newer nurses.

[The purpose of the RRT is….] To get help out where the patients are, to stabilize unstable patients and accommodate patient needs. One might say, you call the RRT when the patient's needs have outgrown the resources available on the ward. Whatever the reason is, if the patient is circulatory or respiratory affected, or due to lack of colleagues in the shift. And if you feel insecure, or you are new. (Focus gr. No. 5)

Ward nurses often activated the RRT when patient needs exceeded ward resources or staff availability. Less experienced nurses were more likely to call the RRT when feeling uncertain about a patient's condition.

### 4.2 Supporting Nurses' Sense of Security

The presence of an RRT‐nurse significantly enhanced nurses' sense of security. This was due to both the readily available safety net and positive interactions with RRT‐nurses. Ward nurses felt reassured when the RRT managed critical situations. Importantly, RRT support not only alleviated immediate pressures but also bolstered nurses' confidence.

It is nice to know that you can always call a competent nurse. I think it also gives a sense of security for new nurses. When they are doing a shift, and we are in each end of the ward with deteriorating patients, you know you always have that lifeline. I think the RRT is a gift! Helping us when we can no longer manage ourselves. (Focus group No. 3)

The RRT was perceived as a valuable resource, especially for less experienced nurses, facilitating both patient stabilisation and their own clinical development. Ward nurses consistently praised RRT‐nurses' professional qualifications, emphasising that strong interpersonal skills were vital for effective collaboration and patient care. They particularly valued RRT‐nurses' empathy, supportive approach and non‐judgmental response to their concerns. The RRT, therefore, was seen as a reliable source of reassurance and practical assistance during patient crises.

I think the RRT is a safety net and RRT‐nurses sense if they are needed to stay like a little longer, when your heart rate is 340… when you have your first evening shift, right? The RRT is a lifeline so that you always… I tell my new colleagues: “You can always just call and ask them anything”. And I think that gives peace of mind. The RRT is a good thing in that way, especially to new nurses. There are lots of nurses who quit their job because they really feel that they are being thrown into deep water. The RRT is perhaps one of the only lifelines you have. (Focus Group No. 1)

RRT‐nurses were consistently described as trustworthy, committed and effective communicators, serving as a vital *lifeline* and *safety net*, especially for new nurses. This reliability provided crucial reassurance, enabling nurses to confidently seek guidance and support during challenging situations. The RRT's prompt intervention and expert assistance were seen as significant factors in preventing burnout and enhancing job satisfaction.

### 4.3 Delivering Practical Assistance and Teaching

Ward nurses utilised the RRT for a range of needs, from complex procedures requiring specialised expertise in circulatory and respiratory issues to basic nursing tasks. These latter calls sometimes stemmed from ward nurses' lack of confidence, perceived skill deficits, or varying interpretations of appropriate RRT utilisation. In situations where ward‐level competence was limited, the RRT‐nurse was the preferred resource for any assistance.

Sometimes when I sit and think back on situations during the dayshift, where I can get practical assistance from other colleagues, I actually think I'd rather have RRT assistance (giggles). (Focus Group No. 7)

Ward nurses recognised the RRT's ability to provide more specialised and immediate support than ward colleagues. Crucially, the RRT facilitated bedside education, instructing nurses on both fundamental and complex clinical procedures.

The RRT is for the good and benefit of the patients and the RRT contributes with greater skills in an emergency situation and can prevent something from happening and we [ward nurses] get guidance and learn to handle future emergency situations. The RRT benefits both us and the patients. There is always learning in a RRT‐review, and there is always communication and training. (Focus Group No. 8)

Participants consistently viewed RRT reviews as valuable training opportunities, fostering the development of basic clinical skills under expert guidance. Consequently, the RRT emerged as a vital resource for both patient care and staff development. By providing immediate, specialised care, the RRT helped prevent adverse patient outcomes and reduce complications, while simultaneously enhancing ward nurses' capacity to manage future emergencies.

### 4.4 Providing Patient‐Centred Care

RRT‐nurses were consistently perceived as prioritising patient well‐being, demonstrating a shared commitment to optimal care. Their ability to maintain perspective, composure and involve both patients and relatives was highlighted as a key strength. Ethical considerations were implicitly embedded in their care. Nurses recognised the RRT's role in initiating challenging end‐of‐life discussions and involving relatives in decision‐making, identifying this as a critical area for departmental improvement.

I am often very inspired by the RRT‐nurses, because no matter what condition the patients are in, they involve them. I think it ([the RRT]) is a small attempt to break down these silos in the hospital, right? The RRT goes completely across the organization. In other words, it is the patient's interests that we look after here. The RRT really put the patient at the center and meet patient needs. (Focus Group No. 2)

The RRT was seen as instrumental in enhancing patient‐centred care, breaking down silos and promoting a holistic approach. RRT nurses were respected for their compassionate and empathetic approach, prioritising patient comfort. By collaborating across departments and disciplines, the RRT demonstrated a clear commitment to placing patients' needs first.

### 4.5 Fostering Constructive Communication and Collaboration

Ward nurses found experienced physicians supportive of the RRT, recognising its role in facilitating ICU transitions and actively participating in RRT reviews. In contrast, nurses expressed greater confidence in RRT‐nurses compared to junior physicians, citing their prompt response, accurate assessments and hands‐on care. Inter‐professional barriers between junior physicians and ward nurses were identified as hindering timely escalation of care. Ward nurses perceived RRT‐nurses as more decisive and proactive, capable of quickly assessing patients, initiating interventions and making critical decisions, thereby empowering ward nurses and improving patient care. However, nurses also expressed frustration with the lack of physician involvement in treatment limitation discussions (LOMTs), highlighting a gap in communication and collaboration.

I've been in situations with junior physicians several times, I don't know if it is about prestige, but junior physicians are not good at calling their senior consultant. Whereas an RRT‐nurse quickly assesses the situation, and goes like that: “Are there any treatment limitations? This patient is too unstable to be on the ward and needs intensive care”. In the past, it was only the physician we could call to decide various things. But now it is the RRT‐nurse who actually takes a position on many things. I often think why do we even call the physician? (Focus Group No. 6)

RRT‐nurses were viewed as effective communicators, utilising the SBAR framework to concisely convey patient information and care plans, which also served as valuable training for ward nurses. Their communication skills, clinical expertise and authority positioned them as a crucial bridge between ward nurses and junior physicians.

It is important to be precise when communicating, you need to introduce the patient, tell what is wrong, what you've done and what you are worried about and what you need help with…… it's like SBAR. Often, I think that the communication and collaboration with the RRT‐nurse is better than… can be better than with a junior physician. And if you are collaborating with an insecure junior physician, then it's not much fun. I need greater skills…yes that's it. (Focus Group No. 8)

Consequently, the RRT was seen as a valuable resource for improving communication and collaboration, particularly when working with junior physicians who may lack experience or confidence.

## 5 Discussion

Understanding ward nurses' perceptions of nurse‐led RRTs is important for improving patient care and safety culture. According to the NHS, a positive safety culture is a dynamic social construct shaped by individual and team interactions [35]. To foster such a culture, it is essential to prioritise relational interactions, positive values, psychological safety, continuous learning and patient‐centred care [35].

This study found that ward nurses highly value the expertise and specialised clinical skills of RRT‐nurses. These findings are supported by previous research by Hession & Meaney (2022), noting the value the RRT brings in managing deteriorating patients and potentially improving patient outcomes [25]. However, there was variability in how nurses defined the RRT's purpose and when to activate it, with some not consistently relying on the EWS algorithm. This aligns with other studies that found a dualism in professional boundaries that could serve as both a facilitator and a barrier to escalation of care [11, 14, 36]. A qualitative evidence synthesis by Ede et al. (2020) explored escalation of care in acute settings. Their findings highlight the crucial role of both objective data, like EWS and subjective cues, such as nurses' observations, in nurses' decision‐making [37]. However, the study also suggests a potential pitfall—a rigid reliance on the EWS algorithm and pre‐defined criteria for calling the RRT could impede timely escalation if nurses hesitate to call outside those parameters [37].

Ward nurses perceived the RRT as a crucial safety net, particularly in high‐pressure situations. They valued the RRT‐nurses' empathy and humility. The interaction between the RRT and those who call the RRT is important, and accountability has been identified as a motivating factor for nurses to activate the RRT, which in return functions as a greatly valued safety net [38]. A systematic review by Hect et al. (2024) defined three key domains influencing RRT activation: ‘hospital infrastructure’, ‘clinician culture’ and ‘nurses' beliefs, attributes and knowledge’. Conversely, a hierarchical or punitive culture might discourage RRT activation due to fear of criticism [39].

The present study also revealed a link between reliance on RRTs for basic procedures and a potential lack of experienced nurses on the wards. This is in line with findings from Chua et al. (2017) and highlights the potential impact of nurse education, skill sets and unit culture on RRT utilisation [19]. Less experienced nurses often activated the RRT due to perceived skill deficits, while experienced nurses used it for consultation and shared decision‐making. This highlights the RRT's significant educational role. However, a study by Bunkenborg et al. found that training ward nurses in basic clinical tasks was a time‐consuming and therefore stressful add‐on for the RRT nurses, as it was regarded as stepping in for experienced ward nurses [40].

Ward nurses consistently valued the RRT's ability to provide patient‐centred care and balance evidence‐based practices with individual patient needs. While our findings align with Engle et al.'s study (2021), which emphasises the importance of a multidisciplinary approach to care, and highlight the importance of providing timely, evidence‐based and patient‐centred care to critically ill patients [41]. However, as Engle et al. point out, evidence‐based practice and patient‐centred care can sometimes represent opposing tendencies [41]. These findings are consistent with previous research on nurse‐led RRTs, which have shown to benefit hospitals by delivering timely care, providing learning opportunities for staff, and breaking down communication barriers to improve overall organisational cohesion [42, 43]. The nurse‐led RRT also played a crucial role in breaking down communication barriers and fostering collaboration, particularly between ward nurses and junior physicians. Ward nurses expressed greater confidence in RRT‐nurses compared to junior physicians, suggesting potential inter‐professional barriers that hindered timely escalation. These findings align with previous research calling for improved collaboration between nurses and physicians [44].

The RRT was seen as a vital resource for improving communication and collaboration. However, the study identified instances where ward nurses bypassed established guidelines and activated the RRT prematurely, possibly due to communication breakdowns. This echoes qualitative research by Dukes et al., (2019) that highlights strong communication and teamwork as essential for fostering a culture where nurses feel comfortable activating the RRT without hesitation [45]. Furthermore, nurses expressed frustration with the lack of physician involvement in LOMTs discussions, highlighting a gap in communication and collaboration. Our study determines the important role of the nurse‐led RRT in identifying patients with clinical deterioration and end‐of‐life care needs, where decision making regarding LOMTs is warranted. Studies have demonstrated that structured communication and interdisciplinary rounds can enhance staff awareness, improve processes and ultimately lead to better patient outcomes [46, 47].

To optimise RRT utilisation and interprofessional teamwork, fostering a strong safety culture characterised by open communication, mutual respect and shared commitment to patient safety is essential [48, 49]. Prioritising staff training and development can further enhance collaborative and effective responses during critical situations [48, 49].

### 5.1 Strengths and Limitations

This study, while offering valuable insights into nurses' perceptions of nurse‐led RRTs, is subject to certain methodological considerations. The focus on hospitals with well‐established RRTs may limit transferability to nascent systems, though it allowed for in‐depth exploration within a mature context. The voluntary participation introduces potential self‐selection bias.

Data collection pre‐dating the COVID‐19 pandemic presents a temporal limitation, necessitating future research to explore pandemic‐related impacts. The predominantly female sample, while reflecting the nursing profession's demographics, may present a limitation in study findings, which needs to be considered when assessing transferability to other settings.

Focus group methodology, while effective, is susceptible to predispositions like dominance effects and groupthink. To mitigate these, the moderator encouraged diverse perspectives, and researcher reflexivity was employed throughout the process to enhance trustworthiness. While some focus groups had smaller participant numbers, data saturation was achieved, aligning with qualitative research principles that demonstrate saturation can occur with fewer participants than often assumed. This ensured rich, in‐depth data collection, providing a comprehensive understanding of nurses' experiences with RRTs.

## 6 Conclusion

This study highlights the significant contribution of nurse‐led RRTs to ward nurses' experiences. Findings reveal the RRT's crucial role in providing timely, non‐confrontational clinical support and mentorship, fostering a secure and collaborative environment. Notably, the RRT effectively mitigates challenges related to understaffing and complex multidisciplinary communication.

The RRT's patient‐centred approach, characterised by calmness and professionalism, creates a supportive environment during critical patient deterioration. While the RRT offers valuable educational opportunities, particularly for novice nurses, it is essential that this complements, rather than replaces, comprehensive unit‐based education and mentorship.

Furthermore, our research underscores the importance of shared decision‐making, especially concerning LOMT, for optimising end‐of‐life care and enhancing patient safety. In essence, nurse‐led RRTs, when effectively implemented, serve as a vital resource for improving both the quality of care and the professional well‐being of ward nurses.

## 7 Clinical Implications

This study underscores the importance of understanding ward nurses' perceptions of nurse‐led RRTs to enhance their effectiveness in improving patient care and safety culture. By gaining insights into nurses' experiences, hospitals can implement strategies to optimise RRTs, such as prioritising staff training, fostering a positive safety culture and promoting shared decision‐making, particularly regarding end‐of‐life care and LOMT.

## Author Contributions

All the authors take full responsibility for this article.

## Conflicts of Interest

The authors declare no conflicts of interest.

## Data Availability

Data cannot be made available due to participant confidentiality.
